# Process mapping in healthcare: a systematic review

**DOI:** 10.1186/s12913-021-06254-1

**Published:** 2021-04-14

**Authors:** Grazia Antonacci, Laura Lennox, James Barlow, Liz Evans, Julie Reed

**Affiliations:** 1 Department of Primary Care and Public Health, Imperial College London, National Institute of Health Research (NIHR) Applied Research Collaboration (ARC) Northwest London, London, UK; 2 Business School, Centre for Health Economics and Policy Innovation (CHEPI), Imperial College London, London, UK; 3 Department of Primary Care and Public Health, Imperial College London, National Institute of Health Research (NIHR) Collaboration for Leadership in Applied Health Research and Care (CLAHRC) Northwest London, London, UK

**Keywords:** Process, Mapping, Health care, Quality, Improvement, Methods, Systematic review

## Abstract

**Introduction:**

Process mapping (PM) supports better understanding of complex systems and adaptation of improvement interventions to their local context. However, there is little research on its use in healthcare. This study (i) proposes a conceptual framework outlining quality criteria to guide the effective implementation, evaluation and reporting of PM in healthcare; (ii) reviews published PM cases to identify context and quality of PM application, and the reported benefits of using PM in healthcare.

**Methods:**

We developed the conceptual framework by reviewing methodological guidance on PM and empirical literature on its use in healthcare improvement interventions. We conducted a systematic review of empirical literature using PRISMA (Preferred Reporting Items for Systematic Reviews and Meta-Analyses) methodology. Inclusion criteria were: full text empirical study; describing the process through which PM has been applied in a healthcare setting; published in English. Databases searched are: Medline, Embase, HMIC–Health Management Information Consortium, CINAHL-Cumulative Index to Nursing and Allied Health Literature, Scopus. Two independent reviewers extracted and analysed data. Each manuscript underwent line by line coding. The conceptual framework was used to evaluate adherence of empirical studies to the identified PM quality criteria. Context in which PM is used and benefits of using PM were coded using an inductive thematic analysis approach.

**Results:**

The framework outlines quality criteria for each PM phase: (i) preparation, planning and process identification, (ii) data and information gathering, (iii) process map generation, (iv) analysis, (v) taking it forward.

PM is used in a variety of settings and approaches to improvement. None of the reviewed studies (*N* = 105) met all ten quality criteria; 7% were compliant with 8/10 or 9/10 criteria. 45% of studies reported that PM was generated through multi-professional meetings and 15% reported patient involvement. Studies highlighted the value of PM in navigating the complexity characterising healthcare improvement interventions.

**Conclusion:**

The full potential of PM is inhibited by variance in reporting and poor adherence to underpinning principles. Greater rigour in the application of the method is required. We encourage the use and further development of the proposed framework to support training, application and reporting of PM.

**Trial Registration:**

Prospero ID: CRD42017082140

**Supplementary Information:**

The online version contains supplementary material available at 10.1186/s12913-021-06254-1.

## Introduction

There is a growing awareness that quality and safety failures in healthcare are attributable more to systems and processes than to human errors [1–4]. To address this, healthcare leaders are increasingly applying quality improvement (QI) and process-oriented management practices from other industries including Lean, Six Sigma, Failure Mode Effects Analysis (FMEA), Failure Mode, Effects, Criticality Analysis (FMECA), and operational research and process-oriented costing approaches such as Time-Driven Activity-Based Costing (TDABC) [5–8].

Applying QI methodology is challenging as healthcare processes are highly variable, distributed and multidisciplinary, involving stakeholders with differing interests and motivations [9–15]. Research shows that the success of QI interventions is heavily influenced by their context of implementation [16–18]. Developing interventions that are adapted to the local context and setting is an essential component of successful QI [19–21], along with engagement of stakeholders [22–24]. Process mapping (PM) has the potential to support QI projects in healthcare by engaging stakeholders to create a shared understanding of the systems they are trying to change [25–29]. However, there is little research on the use of PM in healthcare and whether it is achieving its full potential. The term ‘process mapping’ is used to describe several approaches and techniques. Here we refer to the “entire approach that leads to a holistic understanding of the process under review” [12, 30].

Research shows that the full benefits of PM are accomplished when it is used throughout all the stages of a QI project to plan, implement, monitor and evaluate interventions [12, 29, 31–33]. However, the application of PM within QI initiatives has proved challenging due to the limited time clinicians can devote to it and their limited knowledge of PM methods [34–36].

Although more informed and systematic use of PM in the design and management of healthcare delivery is advocated [37, 38], there is poor evidence on the use of PM and its effectiveness in healthcare [39–41]. To advance current knowledge on PM and improve its use in practice, we need greater insight into how it works in different contexts, the mechanisms underlying its successful use, and challenges to its implementation [42, 43].

There is currently no systematic review of the use of PM in healthcare practice. Most published literature only describes empirical studies of individual interventions using PM. There is very limited information on the range and type of healthcare settings in which PM has been used or the benefits of its use. This problem is compounded by the lack of formal criteria to guide the implementation, evaluation and reporting of PM. Some methodological guides focus on PM in healthcare improvement initiatives [29, 31, 32, 44]; none are based on the structured review of the research evidence. Limited knowledge of the use of PM as a QI method in healthcare hinders its wider adoption [45]. Therefore, increased awareness of its possible applications and benefits, as well as evidence-based quality criteria for its use, are needed.

This paper reviews the empirical literature and methodological guidance on PM to increase understanding of its use in healthcare to: (1) develop a conceptual framework identifying different phases in PM, with quality criteria for each, to guide the effective implementation, assessment and reporting of this method; (2) identify the context of use of PM in healthcare QI projects; (3) assess adherence of the application of PM as described in empirical literature to the proposed conceptual framework quality criteria and (4) explore the reported evidence for the benefits of using PM in improvement work.

## Methods

### Conceptual framework development

**Fig. 1:**
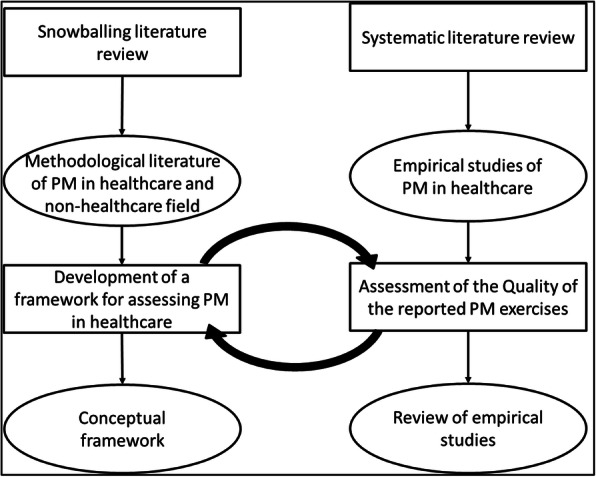
Literature review - Study Method

**Fig. 2:**
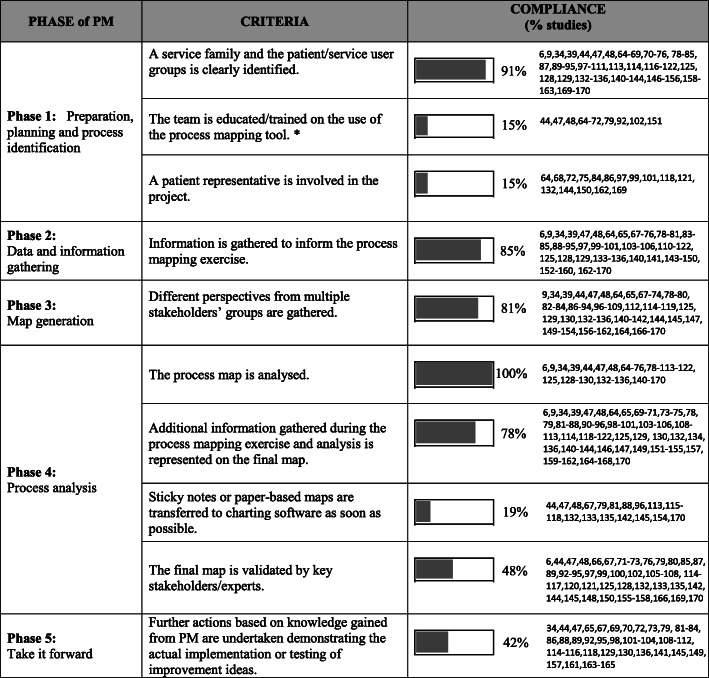
Conceptual framework for process mapping describing a. Phases of PM b. overarching criteria / standards for the PM process (* including 2 cases saying training was not needed as team members already had experience of PM [47, 48])

Given there was limited literature available on the use of PM and little practical guidance on its use in healthcare, we recognised the need to draw on evidence from other relevant fields such as manufacturing and other service industries. We conducted a snowballing review of methodological literature on PM in both healthcare and non-healthcare settings (Fig. 1*,* online supplementary appendix 1). The objective was to identify recommended good practice methods for conducting PM [46]. We identified the most cited studies by searching online databases (Google Scholar, Scopus, Medline, Embase) with keywords derived from our research questions. We then screened citations and reference lists from these sources and included relevant studies. Given the high number of irrelevant articles derived from the online database search, the snowballing technique was very useful to identify the few available methodological studies on PM, as it allowed us to find grey literature, that might be missed by conventional online search methods. We then assessed methodological guidelines (online supplementary appendix 1) and empirical literature selected in the systematic review (2.2, Fig. 1) and through a process of inductive and deductive analysis we developed a conceptual framework identifying overarching quality criteria for each phase of the PM process (3.2, Fig. 2). These criteria were discussed by all authors and selected if they could be applied to a wide range of PM approaches and QI project types. Iterative development of the framework continued as new ideas emerged through discussions and feedback from experts and practitioners.

### Systematic literature review

A systematic literature review of empirical research reporting on the use of PM in healthcare was performed following PRISMA (Preferred Reporting Items for Systematic Reviews and Meta-Analyses) standards [49] and registered on PROSPERO (ID: CRD42017082140).

#### Search and information sources

The search was designed to identify English-language empirical studies describing the use of PM in healthcare. The definition of search strategies was supported by an expert medical librarian. Databases searched were Medline (from 1946), Embase (from 1974), HMIC–Health Management Information Consortium (from 1979), CINAHL-Cumulative Index to Nursing and Allied Health Literature (from 1937), Scopus (from 1960). The last search date was 9 November 2019. Search terms used were “process map*” as a free text search term in title, abstract and keywords for all the selected databases except Scopus, where the search was “process map*” AND “health”. No restrictions on time were imposed on the search.

One author (GA) performed the search and extraction of article references and abstracts.

#### Data collection process and study selection

Inclusion criteria were: full text empirical study; describing the process through which PM has been applied in a healthcare setting; published in English. Methodological studies, posters and conference proceedings were excluded. Articles were first screened by title and abstract by GA. Two reviewers (GA, LL) then independently assessed 20% of full-text articles to test the objectivity of selection criteria, which were then refined. GA and LL independently continued the selection process for the identified articles. Disagreements between reviewers occurred in 4% of cases and were resolved through discussion between all authors.

#### Data extraction

A data extraction form was designed to collect general study information alongside a set of features characterising a PM exercise (e.g. adherence to main criteria for each phase of PM, way in which PM was created, software used to draw the process map, use of online supplements to report complete process maps), from a wide range of projects with different characteristics. The development of the data extraction sheet (online supplementary appendix 3*)* was based on the findings from the snowballing review of literature on PM methodologies and a preliminary screening of all the full text articles included in the empirical research literature review. This initial version was pilot-tested on the 20% of articles (purposively selected to represent different contexts of use of PM) and progressively refined during the data extraction process. Each step in the refinement of the data collection sheet was agreed by all the authors.

Data were extracted from included studies independently by two authors (GA, LL). When disagreements occurred, the other reviewers were involved, and agreement was reached through consensus.

#### Data synthesis and analysis

The analysis was performed independently by two authors (GA, LL) and discussed with the other reviewers. Each manuscript underwent line by line coding. Context in which PM is used (3.3) and benefits of using PM in improvement work (3.5) were identified from the selected studies and coded using an inductive thematic analysis approach [50–52]. Compliance of application of PM to the conceptual framework criteria (3.4) was assessed by coding empirical articles’ adherence to each of the quality criteria and counting the number of studies reporting on the presence of corresponding data item [46]. To ensure accuracy and control for bias in the analysis, all stages of the analysis were progressively discussed by authors and various QI experts and practitioners (researchers, improvement science managers, project managers, and data analysts from the National Institute of Health Research (NIHR) Collaboration for Leadership in Applied Health Research and Care Northwest London (CLAHRC NWL) who were trained in improvement science systematic approaches and tools) [53–57]. Results were summarised using descriptive summaries as well as ratios (for details on the analysis process see online supplementary appendix 2).

#### Quality assessment and risk of bias

The quality of each study and risk of bias were assessed using the Critical Appraisal Skills Programme (CASP) checklist [58, 59]. Two authors (GA, JB) rated the articles independently with disagreements resolved through consensus (see online supplementary appendix 4). CASP was selected because several of the articles included in the review were qualitative studies and covered a wide range of QI projects. Assessing the effectiveness of the projects in which PM has been used was not relevant for this review, therefore results of individual studies were not analyzed. As reported in other qualitative reviews, articles were not excluded or stratified by risk bias [50, 51, 60–63]. Rather, we considered the relative contribution of low/high quality studies in the analysis phase [50, 61]. Moreover, as the review is based on information reported in the selected empirical literature, publication bias as well as bias due to the reporter and the selection of studies may have affected the results of this study *(*see Limitation section).

## Results

### The conceptual framework

Six studies were identified in the snowballing literature review of methodological publications on PM in healthcare and other service and manufacturing industries to develop the conceptual framework (online supplementary appendix 1). The conceptual framework (Fig. 2) described below provides quality criteria for each of the five phases characterizing the process of PM taken from the methodological literature.

(i) *Preparation, planning and process identification*

The service family under analysis and those who will use the process/service (e.g. patients/services users/customers etc.) should be clearly identified and representatives from these groups should be involved in the project. It’s also important that participants have the right skills required to participate in the PM exercise, which might vary depending on the type of project, modelling language and methods used for the analysis. Training should be provided to PM participants to fill skills gaps, if needed. For example, for simple PM exercises this might include a quick introduction to the PM method, while for PM exercises involving more sophisticated approaches (e.g. Six Sigma) and/or more structured modelling languages (e.g. Business Process Modelling Notation), a more technical intensive training might be appropriate.

(ii) *Data and information gathering*

Information should be gathered to inform the PM exercise. In addition to multi-disciplinary meetings, data can be collected using different approaches, such as direct observations, interviews, self-reported patient experiences, analysis of electronic health records or other relevant databases, literature or document analysis.

(iii) *Process map generation*

Different perspectives should be gathered by people having diverse roles in the process, each bringing their view and knowledge of the process under analysis.

(iv) *Analysis*

The process map should be analysed to identify gaps in the systems and opportunities for improvement. The final process map should be checked for accuracy and validated by key stakeholders/experts. During the analysis phase it’s good practice to annotate the process map with information derived from the analysis (e.g. activity durations, resources involved) and transfer paper-based maps in an electronic format. Having a tidy electronic version of the process map supports the analysis and the documentation of the PM exercise and it’s also useful to disseminate and share the map with interested parties or those involved in the process for comments and validation.

(v) *Taking it forward*

Process maps should be used to guide process improvement initiatives. Improvement ideas and actions generated throughout the PM exercise should be implemented to improve current systems and practice.

### General study characteristics

**Fig. 3:**
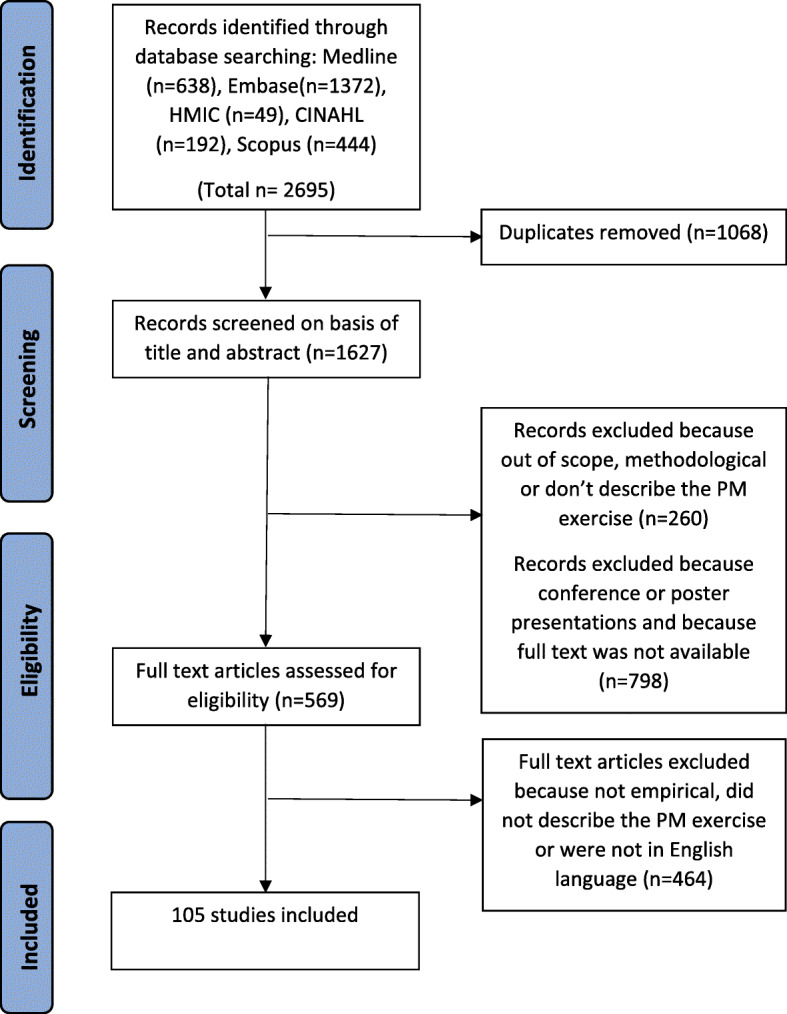
PRISMA Diagram. Description of study selection process

The study selection process for the systematic review of empirical studies using PM is reported in Fig. 3. A total of 105 articles met the inclusion criteria and were included in the review (online supplementary appendix 3). Study quality was moderate-high with 31% study scoring 10/10, 43% scoring 9/10, 20% scoring 8/10 and 6% scoring 6–7/10 (online supplementary appendix 4). 86% were published in or after 2010 and 65% were conducted in the USA and UK (online supplementary appendix 5).

### Context in which PM is used

**Fig. 4:**
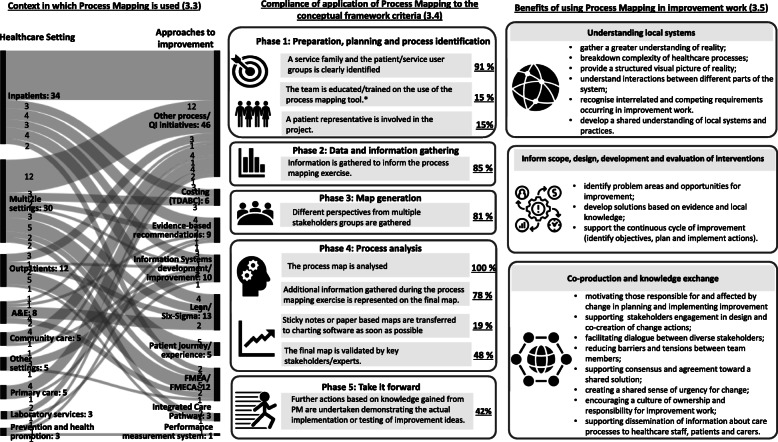
Context in which PM is used, compliance of application of PM to the conceptual framework criteria and benefits of using PM to address complexity of improvement work. (* including 2 cases saying training was not needed as team members already had experience of PM [47, 48])

PM exercises were reported from a wide range of healthcare settings including, *in-patient services* (32%), *multiple settings* (29%), *outpatient* (11%), *A&E* (8%), *community care* (6%), *care provided in other settings* (5%), *primary care* (5%), *prevention and health promotion* (3%) and *laboratory services* (3%). The most common type of projects reporting the use of PM in healthcare were *process improvement/QI initiatives* (68%), which include the use of FMEA/FMECA (11%) and Lean and Six Sigma (12%) approaches. Use of PM in *health information technologies* (HIT) projects was reported in 10% of studies. A few studies outline its use to develop and share *evidence-based recommendations and pathways* (9%). Others reported using PM to identify care process steps within *activity-based costing* methodologies (6%) or to provide a visual representation of *patient journeys (5*%). Only 3% of papers described the use of PM in *integrated care pathway* (ICP) projects. (Fig. 4).

### Compliance of application of PM to the conceptual framework criteria

We assessed all empirical studies against the conceptual framework quality criteria for each phase of PM. The key findings are displayed in Fig. 2.

No study reached overall compliance for all 10 the criteria. Only 7 studies (7%) adhered to either 9/10 (2%) criteria or 8/10 criteria (5%). For five of these seven studies the criteria about involvement of those who would be using the processes (e.g. patient involvement) was not met. Most studies adhered to 7/10 (15%), 6/10 (32%) and 5/10 (35%) of the criteria. The remaining 10% of studies were compliant to 4/10 criteria.

*Phase 1:* Most projects clearly identified a service family and the patient/service user group (91%), but patient representative involvement was only reported in a small number of projects (15%). Team member training in the technique prior the PM exercise was reported in only 15% of projects. This was usually delivered throughout meetings and by using examples [44, 64–70], while in some cases this included intensive QI training [71, 72].

*Phase 2:* 85% of the studies stated that data and information had been gathered to inform the PM exercise as a substitute for (55%), or in addition to (30%), the group knowledge generated in the facilitated process mapping sessions. This included evidence-based best practice recommendations [34–155], interviews [64, 73–148, 154, 156, 158, 160], and more detailed approaches such as observations, operational data collection, time-and-motion studies, and video footage [48, 78–81, 141, 143–146, 150, 159, 163–169].

*Phase 3:* 81% of studies included perspectives from diverse stakeholders. In less than half of the studies (45%), the maps were generated by multiple stakeholder groups; in the remaining studies, maps were generated by researchers. Most projects using industrial engineering approaches created maps in multi-stakeholder meetings (69%).

*Phase 4:* All 105 studies reported that the process map was analysed. How and at what point the analysis was carried out varied significantly, depending on the type of project being reported. Most studies (91%) reported that the map was created to represent current state practice. Four projects [82–85] reported both current state and ideal or future state maps, while 5 describe the ideal or future state process [65, 86–89]. For example, in *TDABC* projects the analysis is focused on process costs and is mainly completed after the current state process map is created [90–95]. In projects implementing evidence-based recommendations, a process analysis was completed before the creation of the ideal or future state process map [89]. Most studies (78%) reported that additional information gathered during the PM exercise - such as delays, safety problems, or flow of information, resources and activity - is represented on the final map. Only 19% of studies reported the use of charting software to draw the process map or to make a tidy version of the paper-based map, and only 48% of studies specified that the PM exercise had been reviewed for accuracy and confirmed by key stakeholders or external experts.

*Phase 5:* 42% of studies reported on the implementation of actions following the PM exercise. The remainder identified process or system issues that needed improvement but did not report taking action.

### Benefits of using PM in improvement work

We identified the benefits of using PM in improvement initiatives described in the reviewed empirical literature and grouped them into three areas: (i) *understanding local systems*, (ii) *inform scope, design, development and evaluation of interventions* and (iii) *co-production and knowledge exchange*. (Fig. 4).

(i) *Understanding local systems*

Studies reported that QI teams gained a more realistic understanding of current practice because PM allowed them to gather knowledge from people directly involved in the process under analysis and provided a visual representation of current or enhanced processes [65, 83, 96, 142]. The studies show that PM is a tool to break-down the complexity characterizing healthcare, by providing improvement teams with a structured picture of complex processes, using information from process stakeholders holding different roles and perspectives [34, 88, 90, 96, 98–100, 140]. Diverse views elicited during PM help improvement teams gain a shared understanding of local practices and underlying systemic issues. For example, PM has been found particularly useful to disaggregate care process and identify costs for each process step [90–95] as well as to understand interactions between different parts of healthcare systems. For example, in ICP projects the use of PM to understand systems helped to improve coordination of care across different settings and networks [86, 87, 101], while in FMEA projects it helped to identify potential systems failures [48, 66–68, 100, 102–107].

(ii) *Inform scope, design, development and evaluation of interventions.*

The identification of actual constraints and opportunities within local systems helped assessment of problem areas and development of improvement solutions grounded in research evidence and local knowledge [65, 76, 79, 81, 92, 96, 108–110, 149, 151]. The use of PM before the implementation of Information Systems (IS) has been shown to support project members with diverse backgrounds achieve a shared understanding of the system and has been reported as crucial for solving design challenges [88, 111–115]. Some studies describe how PM has also been used to assess actual care processes against recognised evidence-based standards [79, 99]. Reviewed studies also show how if used throughout the entire project, PM can play a role in the success of improvement initiatives by supporting continuous improvement. In this respect, PM has been found to be particularly useful for clarifying the scope of projects, targeting the intervention and planning improvement actions [9, 79, 116, 161].

(iii) *Co-production and knowledge exchange.*

PM was reported to be particularly useful to engage and motivate project stakeholders in designing and implementing change. Some studies report that PM supported the effective design of HIT by enhancing the involvement of process stakeholders [88, 111–115]. Studies also describe how greater understanding of different perspectives provided by PM encouraged a culture of ownership and responsibility for improvement work [34, 65, 66, 81, 92, 118, 119]. For example, within ICP projects, PM allowed the clarification and reassessment of the roles and responsibilities within the team [86, 87, 101]. Other studies highlight how participation in PM helped to establish sense of urgency in clinicians regarding patient safety issues, thus enhancing their engagement [81, 92, 157]. Reviewed studies also show that the capacity of PM to facilitate the dialogue between diverse stakeholders helps to smooth barriers and tensions occurring during improvement projects or reach consensus on solutions [65, 66, 71, 73, 81, 92, 103, 118, 119]. For example, some studies reported how PM helped to promote the integration of health services across different settings by developing clinical evidence-based recommendations agreed among different healthcare professionals [74, 86, 87, 89]. Finally, studies describe how PM can be a valuable tool for documenting a care process for further dissemination [170]. This is beneficial, for example, in helping to inform patients and carers about their expected journey [75, 99, 120–122] or to support training and education of healthcare professionals [81, 101, 152, 153, 162].

## Discussion

The use of PM within healthcare improvement projects helps to support understanding of complex healthcare systems and adaptation of improvement interventions to their local context. We reviewed methodological guidance on PM, peer-reviewed empirical literature, and developed a conceptual framework to guide effective implementation, assessment, and reporting of PM in healthcare. We assessed adherence of 105 empirical studies to quality criteria outlined in a newly created conceptual framework. Comparison of methodological guidelines and empirical literature helped to identify common features characterising the use of PM across the selected studies. We also identified reported context of use and benefits of using PM in improvement work.

To our knowledge, this is the first systematic literature review exploring the use of PM in healthcare improvement projects. The review demonstrates that PM is used in projects to improve quality and safety in a wide range of healthcare settings. These projects focus on different QI tools and approaches, and use PM either as a standalone methodology or as a support for other QI methods.

Using the conceptual framework, we found inconsistencies in reporting and in adherence to PM quality criteria. None of the studies adhered to all the criteria and only 7% studies adhered to 8/10 or 9/10 criteria. Assessment of adherence was, however, challenging due to variation in reporting of PM exercises across studies. This is attributable both to the diversity of the contexts for using PM and lack of standardised reporting requirements. Analysis of the reviewed studies suggests that poor adherence with quality criteria reflects not just problems in the reporting of PM, but also the conduct of the method.

Although for most reviewed studies, views of different stakeholders were gathered, only 15% reported the involvement of those who would be using the processes such as patients/ service users/ customers. Moreover, less than half (45%) clearly reported that process maps were generated through multi-professional meetings. This suggests that some benefits of PM may not have been realised in these studies, as failure to engage all stakeholders is unlikely to produce realistic process maps or support successful patient-centred QI initiatives. If PM is conducted without appropriate stakeholder participation, some of the benefits derived from the social interactions, such as empathy between professional groups and agreement for shared solutions, are inhibited [12]. Two of the studies identified in the systematic review reported that the limited involvement of clinical staff was related to the difficultly of relieving them from their daily job [102, 117] but reasons for poor patient involvement should be further investigated [24].

Only 14 of the reviewed studies report training in PM techniques as part of the project. Limited training in PM techniques may explain the lack of discussion or consideration of the process modelling language used to draw the process map in the reviewed studies. This finding confirms previous research stating that most projects in healthcare only use flowchart diagrams, regardless the variety of process modelling techniques and tools available [123]. The choice of modelling language used is important in describing and understanding systems analysed with PM and overlooking these aspects can impact its effective use [124]. Furthermore, training project teams in QI is important not only to improve participants’ technical skills, but also to enhance their engagement in the project [69, 103].

Some studies reported that they had to balance the rigorous use of the PM method with resource and time constraints they had to face in practice [48, 93, 103, 113, 117, 125]. Despite reviewed studies demonstrating poor adherence to the identified PM quality criteria, they describe a number of benefits derived from its use in healthcare improvement projects. This demonstrates the key role played by PM in addressing the challenge of designing and implementing change in complex systems. Using PM in improvement work helps to achieve the strategic principles identified by the Successful Healthcare Improvement from Translating Evidence in Complex Systems (SHIFT-evidence) framework (*act scientifically and pragmatically, embrace complexity, engage and empower*) [54]. The capacity of PM to bring together diverse stakeholder perspectives and provide a visual representation of the system is key to address the complexity which characterizes healthcare processes. Within QI projects, PM helped to provide a shared understanding of the reality of complex systems and facilitated dialogue between team members. This increased engagement of project participants and eased their agreement on common solutions to problems, thus supporting two levers recognised as important for successful improvement in complex systems: knowledge co-production and the definition of shared goals across stakeholders [126, 127].

The use of PM as a monitoring and evaluation tool [9, 12, 64, 119, 128–130] appeared to be out of scope of application by many QI teams. Most of the articles we reviewed focus on use of PM to better understand systems only at the early stages of an improvement initiative or to visualise and disseminate process maps as the “output” of the project. Only 42% of the reviewed studies describe actions undertaken following the PM exercise, suggesting there is still more to know on how PM influences action and impact in overall improvement efforts.

Findings from this literature review show there is still much room for improvement in the use and reporting of PM as a QI method. Limited adherence to recommended practice for PM is a finding consistent with the assessment of fidelity reported for other QI methods [46, 131].

### Implications for practitioners and academics

We unpacked the black box of PM as a QI method and outlined quality criteria to guide its systematic use and reporting. Improving the quality of reporting of PM exercises would enhance transparency, encourage appropriate use of PM in practice, and support the definition of a common language to describe the process of PM [24]. We encourage practitioners and researchers to use and test the validity of our conceptual framework when implementing or reporting PM. We also suggest further development of reporting guidance for PM exercises and their use as a starting point in the design of prospective studies exploring the effectiveness of the method. Our findings show that improvements in reporting are required not only to systematically describe the “process” of PM but also for representation of the process map, as we found that many articles report only a partial or sample representation of the process map developed. Online versions of published articles or online supplements [48, 66, 68, 69, 90, 132–136] could provide more detailed process maps as these are often difficult to display in printed versions of journals. Improvements in the way process maps are represented and reported might increase the effectiveness of PM as a key QI method. For example, annotating the process map with operational (e.g. waiting times, activity durations, waste/ value), cost (e.g. resources required to perform each activity), patient experience or other project data (e.g. areas targeted or changed by various plan-do-study-act cycles), can be helpful to visually identify gaps in the systems and document the process analysis throughout the project. Previous studies also demonstrated that successful implementation of QI initiatives depends not only on the conformance to methodological guidelines, but is greatly influenced by contextual factors (leadership, organisational culture, etc.) [16, 137–139]. Our study has not taken into account the influence of context on PM exercises, because these factors cannot be assessed by analysis of the literature. While the main contribution of this study is in identifying quality criteria to support a more rigorous use and reporting of PM, we encourage practitioners and researchers to consider the influence of contextual factors on the effective use of QI approaches.

### Further research

There is a need for further empirical research to explore the impact of improvement initiative context on practical implementation of PM. We partially explored how PM is used in practice by improvement teams in the NHS in a previous empirical study investigating benefits and success factors of PM in a sample of QI projects [12]. However, most of the projects included in this study [12] used the same methodological approach to PM, (multi-stakeholder meetings to generate the process maps). Further empirical research is needed to test whether our findings hold in QI projects developed by teams using different approaches to conduct the PM exercise, as identified in this literature review (e.g. when PM is used within Six Sigma or Lean approaches). Further literature and empirical research could also explore the representation of process maps in more detail. This would provide a wider perspective on how process activities can be represented and annotated with a variety of information (e.g. value/waste, bottlenecks, constraints, patient experience) and how this can influence the effective use of PM within improvement initiatives.

### Limitations

There are some limitations due to the search process. The database search could have included other search terms such as “process model*”, “process design*” or “system design*”, but the authors agreed that the effort required to screen the resulting records was not justified by the purpose and boundaries of the present study.

A key limitation is due to the fact that the systematic review is based on PM exercises as described in the selected empirical literature and not on the analysis of actual practice. This implies that results might be affected by reporting bias and selection of studies, as well as publication bias. The content of publications heavily depends on what journals accept for publication and on the limited space allowed. Therefore, projects using specific approaches (e.g. TDABC, Lean or IS development) are less likely to present a detailed description of the PM process, compared to other process improvement projects. Successful projects are more likely to be published than studies reporting less successful interventions, which may be equally useful for knowledge generation. Bias could also arise because we only searched English-language papers. However, our objective was not to perform an exhaustive review of all the studies applying PM techniques in healthcare, nor to assess the effectiveness of PM, but to provide a representative overview of the use of PM as reported in empirical literature.

Another limitation is due to the fact that PM exercises were usually reported as a part of a wider project. Clearly distinguishing the component attributable to PM from that associated with the whole project was therefore not always straightforward. We addressed this limitation in the development of the data item sheet and the conceptual framework, as well as in the data collection and analysis phase. For example, we decided not to quantitatively assess the different roles involved in the PM exercise, because it was not always clear if and how all team members were involved in the PM exercise. Furthermore, we evaluated the actual implementation of the recommendations derived by the PM exercise, considering the improvement actions reported in respect of the whole project.

Finally, within the included studies we found three papers [120–122] which seemed to derive from the same project. We addressed this bias in the analysis and summary phase by discounting the patterns emerging from common characteristics of these three studies.

## Conclusions

PM is at the heart of a range of different improvement projects in healthcare. Its effective use is often a fundamental component of successful QI initiatives. If appropriately used, PM brings together perspectives of diverse stakeholders to harness tacit knowledge and understand complex processes, as well as to find common solutions and enhance team engagement. However, variance in reporting and lack of compliance with guiding principles underpinning its effective use may inhibit its full potential in healthcare improvement initiatives, and in sharing learning between initiatives. Greater scientific rigor in the application and reporting of PM is required to increase its effectiveness as a method for improvement and advance the field of improvement science.

The conceptual framework proposed in this paper provides generalisable quality criteria to help “unpack the black box” of PM across a variety of settings and problems in healthcare. We encourage the use and further development of these criteria to guide future adoption of PM and for reporting and evaluating its efficacy. A better understanding of the circumstances surrounding decisions about deployment of mechanisms supporting QI methods, such as PM, is needed in order to increase their effectiveness. Greater recognition of the benefits of PM, as well as training in this method for healthcare professionals and improvement leaders would also contribute to its more extensive and appropriate use in practice.

## Supplementary Information

**Additional file 1: Supplemental_Material_1.** Online supplementary appendix 1, Methodological studies selected in the snowballing search. Description of data: details on the methodological studies used to develop the conceptual framework.

**Additional file 2: Supplemental_Material_2.** Online supplementary appendix 2, Systematic literature review - Analysis process. Description of data: description of the data analysis process.

**Additional file 3: Supplemental_Material_3.** Online supplementary appendix 3 – Analysis codes and data extraction details. Description of data: description of the analysis codes and data extraction sheet with the details of data extracted for each article included in the systematic review.

**Additional file 4: Supplemental_Material_4.** Online supplementary appendix 4, Quality assessment using the Critical Appraisal Skills Programme (CASP) checklist. Description of data: rating of each article included in the systematic review according to the CASP checklist’s items.

**Additional file 5: Supplemental_Material_5.** Online supplementary appendix 5, Characteristics of empirical studies. Description of data: general characteristics of the studies included in the systematic review.

## Data Availability

All data generated or analysed during this study are included in this published article [and its supplementary information files].

## Abbreviations

CASP: Critical Appraisal Skills Programme
CLAHRC: Collaboration for Leadership in Applied Health Research and Care
FMEA: Failure Mode Effects Analysis
FMECA: Failure Mode, Effects, Criticality Analysis
HIT: Health Information Technologies
ICP: Integrated Care Pathway
NIHR: National Institute of Health Research
PM: Process Mapping
PRISMA: Preferred Reporting Items for Systematic Reviews and Meta-Analyses
QI: Quality Improvement
TDABC: Time-Driven Activity-Based Costing
