# Supplementary material for: Process mapping in healthcare: a systematic review
Source: BMC Health Serv Res. 2021 Apr 14;21:342. doi: 10.1186/s12913-021-06254-1 (PMC8048073; doi:10.1186/s12913-021-06254-1)
Supplement: Supplementary file 1 — Additional file 1: Supplemental_Material_1. Online supplementary appendix 1, Methodological studies selected in the snowballing search. Description of data: details on the methodological studies used to develop the conceptual framework. [file 12913_2021_6254_MOESM1_ESM.docx]

| **Online supplementary appendix 1**  **Methodological studies selected in the snowballing search** | | | | | | |
| --- | --- | --- | --- | --- | --- | --- |
|  |  | **1. PREPARATION, PLANNING AND PROCESS IDENTIFICATION** | **2. DATA AND INFORMATION GATHERING** | **3. PROCESS MAP GENERATION** | **4. ANALYSIS** | **5. TAKING IT FORWARD** |
| **OTHER INDUSTRIES** | **Jacka, J. M., & Keller, P. J. (2009). Business process mapping:. Improving Customer Satisfaction. Canada.** | Process identification: learning what makes up the process under review assuming an external perspective  • Identify the trigger events • Identify the customer critical processes • Identifying the supporting processes • Name the process • Prepare the broad overview process map  • Identify process measures focused on the customer | • Understand what information you need and where you go to learn it  • Hold meetings with the unit owners and process owners at various levels • Develop data gathering plans  • Use data gathering tools (e.g. Process Description Overview; Process Owner/Unit Owner Chart; Process Profile Worksheet; Workflow Surveys) | Map generation • Build the map in real time using the sticky-note technique  • The final product should look deceptively simple and easily understandable  • Use levels (units, tasks, and actions) to gain an understanding of a process at varying depths  • Use drill-down maps when you need to explore more detail, and create overview maps to summarize. • Visualize the chosen metrics in the process map in a way that serves the purpose of the process map. | • Make sure that the final analysis ties in with the initial assumptions (triggers, inputs, outputs, process ownership, business objectives, business risks, key controls, and measures of success).  • Ensure that map is complete, remove extraneous approvals, looping errors, eliminate or streamline delays, rework and handoffs, review forms and hold files, ensure that map is complete and closely look at cycle times. • Individuals interviewed for the maps, supervisors and managers involved, should have a final look at the maps to ensure that they are correct.  • Obtain the process owner’s buy - in | Share the finalized version of the process mapping and results with all participants (not only with the managers) |
|  | **Damelio, R. (2011). The basics of process mapping. CRC Press.** | Before beginning the session • Provide a clear and compelling goal for creating the map • Select the right people to get in the room: the natural work group • Provide hand-outs showing naming conventions and example action verbs and “just in time” learning • Always walk the process • Establish ground rules upfront and Post them on a Flipchart (e.g. map creation methods and conventions, encourage communication, go for quantity of information – breadth versus depth - , etc.) • Use a room large enough so that people can easily move around • Keep a kit of supplies handy, include plenty of “wall” paper to write on | Five methods to obtain the knowledge necessary to create a process map:  • Self-generate  • One-on-one interview • Group facilitation • Content (document) review • Observation Use the natural workgroup, along with the Observation and Group facilitation methods | During the Mapping work session  • Use “sticky notes” to generate initial activities, etc. • Arrange sticky notes into an initial layout of the process • Make multiple passes through the work sequence as needed, in light of the goal for creating the map • Keep a steady pace, monitor participation and group dynamics • Document the map using digital camera to capture images of the “raw” map, use software to create multiple views of the map to support analysis and interpretation (e.g. wastes and barrier to flow views, Trigger/Priority/Synchronization view, Resource deployment view) | Analysis  • Identification of the serial pattern of the workflow, waste and barriers to flows which cause work to take longer, cost more and produce less • Use of some type of systematic approach to assess a workflow • Use of measures of flow and waste to quantify workflow performance (e.g. value creating time, lead time, etc.) | Use of the 7 principles to shorten lead-time, reduce cost, and increase productivity:  1. Improve flow from the outside in 2. Measure what matters to the customer 3. Make the end-to-end (flow) visible 4. Identify and remove barriers to flow 5. Connect and align value-added work fragments 6. Organize around the end to end flow 7. Manage the flow visually |
| **HEALTHCARE LITTERATURE** | **NHS Institute for Innovation and Improvement (2005). Improvement Leaders' Guide. Process Mapping, analysis and redesign. General improvement skills. NHSI.** | Getting started • Gain the support of key people and  • Define the key roles (sponsors, project leader, champions) Organizing an event to map the patient journey • identify the patient group(s) whose care would benefit most by redesign  • identify the staff groups involved  • organise the event for one full day, or for two half-days  • arrange a suitable venue • gain participants’ engagement (explaining to participants project scope, their roles and their expected contribution) | Data collection is suggested to validate team “guestimates” and further analyse the process by:  • measuring relevant operational data (e.g. times)  • shadowing a real patient  (more information is provided in [www.institute.nhs.uk/improvementguides](http://www.institute.nhs.uk/improvementguides)) | Running the event • Get the lead clinician or the senior manager to attend and preferably to chair or open the event • An independent facilitator is really useful.  • Create an environment which people find safe in order to encourage honesty • Don’t be tempted to try and solve the problems until you have fully mapped the process make the event practical, visual and fun. • Agree the next steps before the event finishes  Mapping the journey • define and agree the group of patients to be mapped and the scope • identify all staff groups involved and map that stage  • record on Post-it notes or draw on flip charts ‘ each step (who does what to the patient) • ensure involvement • defining and mapping the process should take about 60 minutes | Ask • is the patient getting the most appropriate care? • is the most appropriate person giving the care? • is the care being given at the most appropriate time? • is the care being given in the ideal place? **Analyse by identifying** • number of steps in process • number of steps that do not add value to the patient • steps where patients have to wait **Redesign by considering** • changes they would like to test • encourage teams to share possible changes and consider if these could be adapted for their own process | Follow up • Validate process map and analysis outputs with all stakeholders • Plan how to measure and have feedback about actual data • Plan how to monitor that agreed improvement actions are effectively undertaken  Test the process on users (organize a patient group) Work to match capacity and demand and reduce variation particularly at the bottlenecks |
|  | **Trebble, T. M., Hansi, N., Hydes, T., Smith, M. A., & Baker, M. (2010). Process mapping the patient journey through health care: an introduction. BMJ, 341(7769), 394-397.** | Preparation and planning • Form a team of four or five key staff • Determine condition or intervention requiring pathway redesign • Agree aims of project and identify evidence base • Agree team members’ roles, methods, time frame, and locations • Producing a rough initial draft of the patient journey  • Review literature or questionnaire studies of patient’s expectations and outcome | Data Collection Different techniques can be used for data collection, a combination of approaches can be applied. The commonly used technique of walking the patient journey includes interviews with patients and staff and direct observation of the patient journey and clinical environment. It allows the investigators to “see” the patient journey at first hand. | Mapping the information • The process map should comprehensively represent the patient journey. • It is common practice to draw the map by hand onto paper (often several metres long), either directly or on repositionable notes.  • Information relating to steps or representing movement of information (request forms, results, etc.) can be added. • It is useful to obtain any missing information at this stage, either from staff within meeting or by revising the clinical environment. | Collecting missing data and analysis The map can be analysed by using a series of simple questions  • How many steps are involved? • How many staff-staff interactions (handoffs)? • What is the time for each step and between each step? • What is the total time between start and finish (lead time)? • When does a patient join a queue, and is it a regular occurrence? • How many non-value steps are there? • What do patients complain about? • What are the problems for staff?  The additional information can be added to the process map for visual representation. | • Redesign the patient journey • Implement the pathway • Repeat the process mapping exercise |
|  | **Jackson, T. L. (2013). Mapping clinical value streams. CRC Press.** | • Define the value that the process delivers to the patient by specifying a service family • Identify the patient • Identify the team (people involved in the process delivery, patient, external people –“outside eyes”) • Plan the meeting (book a room which is big enough, prepare the supporting material, etc.) | Walk the value stream and Gather data Walk the patient’s journey and collect data using tools such as time observation forms and standard work sheets | Build and analyse the current state map - Value stream mapping workshops (analysis starts while building the map) • Identify sequential operations in the process • Identify operational metrics • Identify waits between operations • Document hoe work is prioritized  • Identify Manual and electronic information flows • Identify external patient flows • Build a process timeline and calculate summary statistics Identify systematically which activities are value-added and which are waste | Brainstorm and build a future-state map that establishes flow wherever possible and – where flow is not possible – links islands of flow together with a pull-system. Guidelines: • Produce services to takt-time • Eliminate unnecessary waits and create clinical cells • If you cannot flow, pull • Send the patient schedule to only one operation (pacemaker) in the process and then pull the patient from upstream processes to the pacemaker • Level the volume • Level the case-mix A future state map describes the same process, but with waste removed. | Implement and perfect improvements • Break the value stream down into implementation “loops” or projects that can be managed separately, either in concert or in a series of projects • For each loop draft a process charter called an A3-T, reporting: o A description of the problem or opportunity for improvement o Quantitative targets for lead-time reduction and quality improvement o A cogent analysis of the causal mechanisms underlying the problem o An outline of the proposed solution o A project timeline together with responsibilities for improvement activities o A method for checking or monitoring progress and publishing results to your organization • Each A3-T comprises a complete cycle of PDCA |
|  | **McLaughlin, N., Rodstein, J., Burke, M. A., & Martin, N. A. (2014). Demystifying process mapping: a key step in neurosurgical quality improvement initiatives. Neurosurgery, 75(2), 99-109.** | In preparation for the process-mapping activity • Define quality improvement project goals • Build a dedicated team and coordinate meetings • Choose a meeting facilitator • Educate the team on the process-mapping tool • Decide on process-mapping medium (paper, white board, mapping software) |  | Process mapping activity • Creating a visual representation of the current state of the process • Set clear boundaries for maps (start and stop points) • Use simple symbols, content construction, connectors, and colours to enhance visual representation • Determine the level of detail needed (macro vs. micro maps) | Correction and approval of process map • Correct the process map with the team members  • Identify key team members to approve corrected maps and integrate corrections to the final maps • Using the process maps to guide process improvement initiatives • Educate the team on process map analysis – going beyond colours and geometry | Using the process maps to guide process improvement initiatives • Identify improvement opportunities • Prioritize improvement opportunities  • Develop an action plan (tools and template depending on quality improvement methodologies) |
