# Supplementary material for: Process mapping in healthcare: a systematic review
Source: BMC Health Serv Res. 2021 Apr 14;21:342. doi: 10.1186/s12913-021-06254-1 (PMC8048073; doi:10.1186/s12913-021-06254-1)
Supplement: Supplementary file 2 — Additional file 2: Supplemental_Material_2. Online supplementary appendix 2, Systematic literature review - Analysis process. Description of data: description of the data analysis process. [file 12913_2021_6254_MOESM2_ESM.docx]

**Online supplementary appendix 2**

**Systematic literature review - Analysis process**

*Context in which PM is used* (3.3)

Healthcare settings and approaches to improvement in which PM has been used were identified from the selected studies using an inductive thematic analysis approach.[49–51] GA and LL developed preliminary codes by coding each line of the text of all of the included studies. This preliminary coding structure was iteratively revised with new constructs integrated and refined as new studies were added to the dataset. These codes were further revised and grouped into broader categories after discussion with all authors and rechecking of the original studies.

*Compliance of application of PM to the conceptual framework criteria (3.4)*

The selected empirical studies were then assessed against the quality criteria for each stage of PM using the conceptual framework (3.2, Table 1). GA and LL coded the selected empirical studies’ adherence to the quality criteria in the framework (3.3). Results were synthetized by counting the number of studies reporting on the presence of the corresponding data item. Studies where the assessed information was missing were excluded*.*[47]

*Benefits of using PM in improvement work* (3.5)

The benefits of using PM were inductively explored through a thematic analysis of the reviewed empirical literature.[49–51] GA and LL developed a set of initial themes inductively by coding included studies. These themes were progressively refined and grouped to generate ‘descriptive themes’. To highlight the contribution of PM to the success of QI initiatives, PM benefits were conceptualized (‘analytical themes’) using as lenses the Successful Healthcare Improvement from Translating Evidence in Complex Systems (SHIFT-evidence) conceptual framework.[53] This describes the constant iterative processes required for effective evidence translation within complex healthcare systems. GA and LE independently analysed the identified themes against the SHIFT framework, and consensus was reached through discussion with all authors and QI experts on the final conceptualization of PM benefits.

Data extraction details and codes used are available on the *Online supplementary appendix 3.*
