# Supplementary material for: Process mapping in healthcare: a systematic review
Source: BMC Health Serv Res. 2021 Apr 14;21:342. doi: 10.1186/s12913-021-06254-1 (PMC8048073; doi:10.1186/s12913-021-06254-1)
Supplement: Supplementary file 5 — Additional file 5: Supplemental_Material_5. Online supplementary appendix 5, Characteristics of empirical studies. Description of data: general characteristics of the studies included in the systematic review. [file 12913_2021_6254_MOESM5_ESM.docx]

**Online supplementary appendix 5**

**Characteristics of empirical studies**

**Year of publication**

2000 - 2005: 6 (5.7 %)

2006 - 2010: 16 (15.2 %)

2011 – November 2019: 83 (79.0 %)

**Country**

USA: 46 (43.8 %)

UK: 22 (21.0 %)

Canada: 6 (5.7 %)

Australia: 5 (4.8 %)

Ireland: 3 (2.9 %)

Brazil: 2 (1.9 %)

Denmark: 2 (1.9 %)

Ghana: 2 (1.9 %)

Qatar: 2 (1.9 %)

Spain: 2 (1.4 %)

Multiple countries: 2 (1.9 %)

Ethiopia: 1 (1.0 %)

France: 1 (1.0 %)

Germany: 1 (1.0 %)

India: 1 (1.0 %)

Israel: 1 (1.0 %)

Jordan: 1 (1.0 %)

Kuwait: 1 (1.0 %)

Malawi: 1 (1.0 %)

Netherlands: 1 (1.0 %)

Portugal: 1 (1.0 %)

South Africa: 1 (1.0 %)

**Healthcare setting**

Inpatient: 34 (32.4 %)

Multiple settings (2 or more): 30 (28.6 %)

Outpatient: 12 (11.4 %)

A&E: 8 (7.6 %)

Care provided in other settings: 5 (4.8 %)

Community care: 5 (6.1 %)

Primary care: 5 (4.8 %)

Prevention and health promotion: 3 (2.9 %)

Laboratory Services (histopathology, microbiology, cytopathology): 3 (2.9 %)

**Type of projects/Overall approach**

Process improvement/ QI initiatives: 71 (67.6 %)

Other Process improvement/QI initiatives: 45

Failure mode and effects analysis (FMEA)/Failure mode, effects and criticality analysis (FMECA): 12

Lean: 8

Six - Sigma: 3

Lean- Sigma: 2

Process simulation: 1

Information Systems (IS): 10 (9.5 %)

IS Design/Development/Implementation: 6

Examination of the impact of Health information technologies (HIT): 2

Understand information dynamics and implication for IS (how ICT can improve processes): 2

Evidence-based recommendations/ care pathways/ clinical decision-making: 9 (8.6 %)

Costing – Time-driven activity-based costing (TDABC): 6 (5.7 %)

Patient journey/ patient experience: 5 (4.8 %)

Integrated care pathway (ICP): 3 (2.9 %)

Development of a performance measurement system: 1 (1.0 %)
